# What influences the decision by primary care doctors to recommend cancer screening? A qualitative evidence synthesis

**DOI:** 10.1136/bmjopen-2025-109989

**Published:** 2026-05-24

**Authors:** Mila Nu Nu Htay, Mon Mon Thawda Oo, Tin Tin Su, Michael Donnelly

**Affiliations:** 1 Centre for Population Health (CePH), Department of Social and Preventive Medicine, Faculty of Medicine, Universiti Malaya, Kuala Lumpur, Malaysia; 2 Centre for Public Health, Queen’s University Belfast, Belfast, Northern Ireland; 3 Faculty of Medicine, Manipal University College Malaysia, Melaka, Malaysia; 4 Jeffrey Cheah School of Medicine and Health Sciences, Monash University Malaysia, Bandar Sunway, Malaysia

**Keywords:** Early Detection of Cancer, Cancer, Primary Care, Guideline Adherence, Decision Making

## Abstract

**Abstract:**

**Objectives:**

This review synthesised evidence from studies about the perceptions and experiences of primary care doctors (PCDs) regarding the factors that influenced or inhibited their decision to recommend cancer screening.

**Design:**

Qualitative evidence synthesis (QES) following the ENTREQ (Enhancing Transparency in Reporting the Synthesis of Qualitative Research) guideline.

**Data sources:**

MEDLINE, Web of Science, Embase and the Cochrane Library for eligible studies that were published between 1 January 2000 and 22 January 2025.

**Eligibility criteria:**

We included peer-reviewed studies which involved PCDs (in private or public settings in any country), covered cancer screening guideline recommendations and written in English.

**Data extraction and synthesis:**

Two independent reviewers conducted article screening, data extraction, quality assessment and coding. The review team discussed, reviewed, and refined the descriptive themes and analytical themes to reach higher-level interpretation.

**Results:**

Nine studies were included in this QES. The synthesis of evidence identified three main analytical themes. First, PCDs demonstrated positive attitudes towards cancer screening guidelines even though there were some concerns regarding false-negative results and colonoscopy-related complications as well as a perceived lack of rewards/compensation for adhering to screening guidelines. Second, implementation challenges were noted including patient factors, PCD-related inhibitors, health system-related barriers and challenges related to cancer awareness and beliefs. Thirdly, the factors that were perceived by PCDs to facilitate guideline adherence included integration of digital record systems, reminders and raising community awareness about screening. Only one study was conducted in a low-middle-income country, and it indicated that resource limitations, including the unavailability of FOBT tests, were reported to reduce PCDs’ motivation to implement cancer screening recommendations. In addition, cancer-related stigma, cultural beliefs and misconceptions about cancer—such as preferring home or religious remedies—were perceived as barriers to screening uptake, highlighting the need for stronger public health education and awareness initiatives.

**Conclusion:**

There tends to be good adherence to screening guidelines among PCDs across various countries and healthcare systems at least relating to colorectal cancer and, to a lesser extent, cervical cancer. Studies about adherence to BC screening in primary care are required. The incorporation of an array of interrelated factors appears to facilitate adherence. We know less about cancer guideline adherence in resource-constrained settings, and there is a need for studies in primary care in LMICs.

**STRENGTHS AND LIMITATIONS OF THIS STUDY**

- This study followed a systematic approach to qualitative evidence synthesis of primary care doctors’ perceptions and experiences regarding their decision to recommend cancer screening.
- Multiple databases (MEDLINE, Web of Science, Embase and Cochrane Library) were searched comprehensively with predefined criteria, enhancing the rigour of study identification.
- The methodological quality of included studies was appraised using the CochrAne qualitative Methodological LimitatiOns Tool (CAMLOT), and confidence in the synthesis findings was assessed using the CERQual approach.
- Independent screening and data extraction were conducted by at two researchers to minimise bias.
- The review included only studies published in English, and the majority of available evidence came from high-income countries, which may limit transferability of findings to low- and middle-income country contexts. The review did not include a search of national screening programme websites, Ministry of Health websites or grey literature.

## Introduction

 Cancer is a major public health challenge, accounting for one-fourth of global non-communicable disease-related deaths^1^ with projections of 20 million new patients in 2022, to 35 million by 2050.^2^ Addressing this challenge requires improved prevention such as vaccination for human papillomaviruses (HPV), tobacco control, early detection and treatment, and palliative and survivorship care.^3^ Screening is key to reducing the burden^4^ yet approximately half of cancer cases are detected late.^5^ Early cancer detection requires resources, technical support, training and locally tailored policies.^6^ WHO recommends breast, cervical and CRC cancer screening programmes^7^ such as HPV DNA testing for cervical cancer from age 30 every 5–10 years.^8^ The US Preventive Services Task Force (USPSTF) advises a biennial mammogram for women aged 40 to 74^9^; and Faecal Occult Blood Test (FOBT) or colonoscopy for adults aged 50 to 75 years.^10^ Screening guidelines vary by resource availability and feasibility.^11^

Primary care doctors (PCDs) are expected to play a valuable role in cancer prevention and early detection as they are the first point of contact in most healthcare systems.^12^ The role of PCDs includes promoting cancer awareness, identifying high-risk patients, screening, referrals and managing follow-up.^13 14^ PCDs tend to fulfil the role of ‘gatekeeper’ in their respective country’s health system and, in relation to this role, initiate cancer screening discussions and, when appropriate, recommend screening tests.^15^ Indeed, a doctor’s recommendation is one of the strongest predictors of patient participation in cancer screening programmes.^16 17^ However, there appears to be considerable variation regarding the implementation of screening in primary care. For example, 12.5% and 60.6% of doctors in Poland ‘routinely’ or ‘sometimes’ informed patients about cancer screening and risks while less than half discussed CRC (31%), cervical (41%) and breast (44%) screening.^12^ This screening practice variation may reflect the challenges faced in a primary care setting such as lack of time and lack of financial resources to provide and deliver cancer prevention services.^12^ Previous research including reviews that examined the factors influencing adherence to cancer screening guidelines focused mainly on provider-patient communication patterns and specific age groups.^1217^,^19^ To the best of our knowledge, there is no qualitative evidence synthesis of primary care doctors’ experiences and perceptions regarding their decision to recommend breast, cervical and colorectal cancer screening across age groups. Thus, this ENTREQ-guided review synthesised studies about PCDs’ views regarding their decisions to recommend breast, cervical and colorectal cancer screening in order to derive insights into cancer screening in primary care, including the use of guidelines and related implementation challenges.^20^

## Methodology

### Database search

A systematic search was conducted in MEDLINE, Web of Science, Embase and the Cochrane Library for studies published in English from 1 January 2000 to 22 January 2025. Ministry of Health websites and reports, national screening programme websites and grey literature were not searched. The search terms were developed by the research team based on domains relating to (1) study population, (2) type of cancer (colorectal, breast and cervical cancer), (3) screening and (4) compliance and adherence to recommended screening guidelines (see online supplemental appendix A, table 1). The details of search strategies can be found in online supplemental appendix C.

### Inclusion and exclusion criteria

Peer-reviewed studies were selected based on prespecified criteria: written in English, published between January 2000 and January 2025, involved PCDs (in private or public settings in any country) and covered cancer screening guidelines. Doctors practising in specialty clinics/hospitals (such as obstetrics and gynaecology clinicians, surgeons, oncologists and radiologists) and nurses, allied health personnel and volunteers in healthcare sectors or NGOs were excluded; studies about patient adherence to screening were excluded. All qualitative designs and qualitative components of mixed-methods studies were included; study protocols and abstracts, national guidelines and systematic reviews were excluded.

### Selection of studies

The search results were exported to COVIDENCE, a software platform for managing systematic review screening^21^ and duplicates were removed. Members of the review team (MNNH, MMTO) paired up and screened the articles independently. Each title, abstract and full text was reviewed by two independent reviewers (MNNH, MMTO) and discrepancies were resolved through discussion with a third reviewer (MD). Any disagreements were discussed and resolved by the review team (MNNH, MMTO, MD, TTS).

### Data extraction

A data extraction form was created based on the review objectives, relevant study information and methodology. The data extraction form (in Excel sheet mode and Microsoft Word document) was piloted using the two eligible papers, and then it was used for the remaining eligible papers. Data from the included papers about study characteristics, methodology and summary of findings were extracted onto an Excel sheet. Data regarding themes, findings, direct quotations, relevant tables, figures and conclusion sections were extracted onto a Microsoft Word document. Data extraction was conducted by two independent researchers (MNNH, MMTO).

### Data synthesis

Extracted data in Word were imported into NVivo software (Version 14). Following the Enhancing Transparency in Reporting the Synthesis of Qualitative Research (ENTREQ) guideline, data were synthesised using the Thomas and Harden’s thematic synthesis approach.^22^ This synthesis followed three steps: (1) coding of the extracted texts, (2) developing descriptive themes and (3) developing analytical themes. Two researchers coded data independently, compared results and agreed on final codes. This method was chosen because of its inductive theme development feature and its ability to reflect the synthesised findings of primary studies.^23^ Coding began with the first study, with other studies mapped to existing codes or newly created ones when needed. Comparison and review of coding were made within and across studies. Sub-themes and themes were developed after two reviewers coded independently (MNNH, MMTO). Next, reviewers discussed, reviewed, and refined the descriptive themes and analytical themes to reach higher-level interpretation.

### Methodological assessment

The methodological strengths and limitations of eligible primary studies (n=9) were assessed using the CochrAne qualitative Methodological LimitatiOns Tool (CAMLOT).^24^ CAMLOT evaluates potential methodological concerns in qualitative studies. The overall concern level about the studies was classified as: ‘no’, ‘minimal’, ‘minor’, ‘moderate’ or ‘serious’. Two researchers (MNNH, MMTO) independently conducted the assessment, and any discrepancies were resolved through discussion.

### Assessment of confidence in synthesis

The review findings were assessed for methodological limitations, coherence, adequacy and relevancy using Confidence in the Evidence from Reviews of Qualitative research (CERQual) approach,^25^ which evaluates confidence in findings. Methodological limitations (Component 1) of included studies was assessed using CochrAne qualitative Methodological LimitatiOns Tool (CAMLOT).^24^ Coherence (Component 2) of findings from the primary studies (n=6) and qualitative component of mixed methods studies (n=3) was assessed in terms of clarity and cogency between data. Adequacy (Component 3) was assessed based on richness and quantity of supporting data. Relevancy (Component 4) was assessed according to the study population, setting and phenomenon of interest to the synthesis question. Two researchers (MNNH, MMTO) conducted the assessment, resolving discrepancies through discussion.

### Reflexivity

The review team consisted of academic researchers in public health, medicine and health sciences in a middle-income Southeast Asian country except for one member from a designated high-income country. Team members had experience of projects related to cancer screening, community health promotion programmes and primary care services. Collectively, prior experiences facilitated an understanding about the role of primary care doctors and the context of cancer screening and early detection. To enhance reflexivity and transparency regarding potential bias related to prior experiences of the review team, team members engaged in critical friend discussions throughout the review particularly during data synthesis, interpretation and theme development. The synthesised findings were grounded in the data of the primary studies rather than reviewers’ prior assumptions.

### Patient and public involvement

Time and resources were insufficient to involve patients or the public meaningfully in the review.

## Results

### Search and study selection

**Figure 1:**
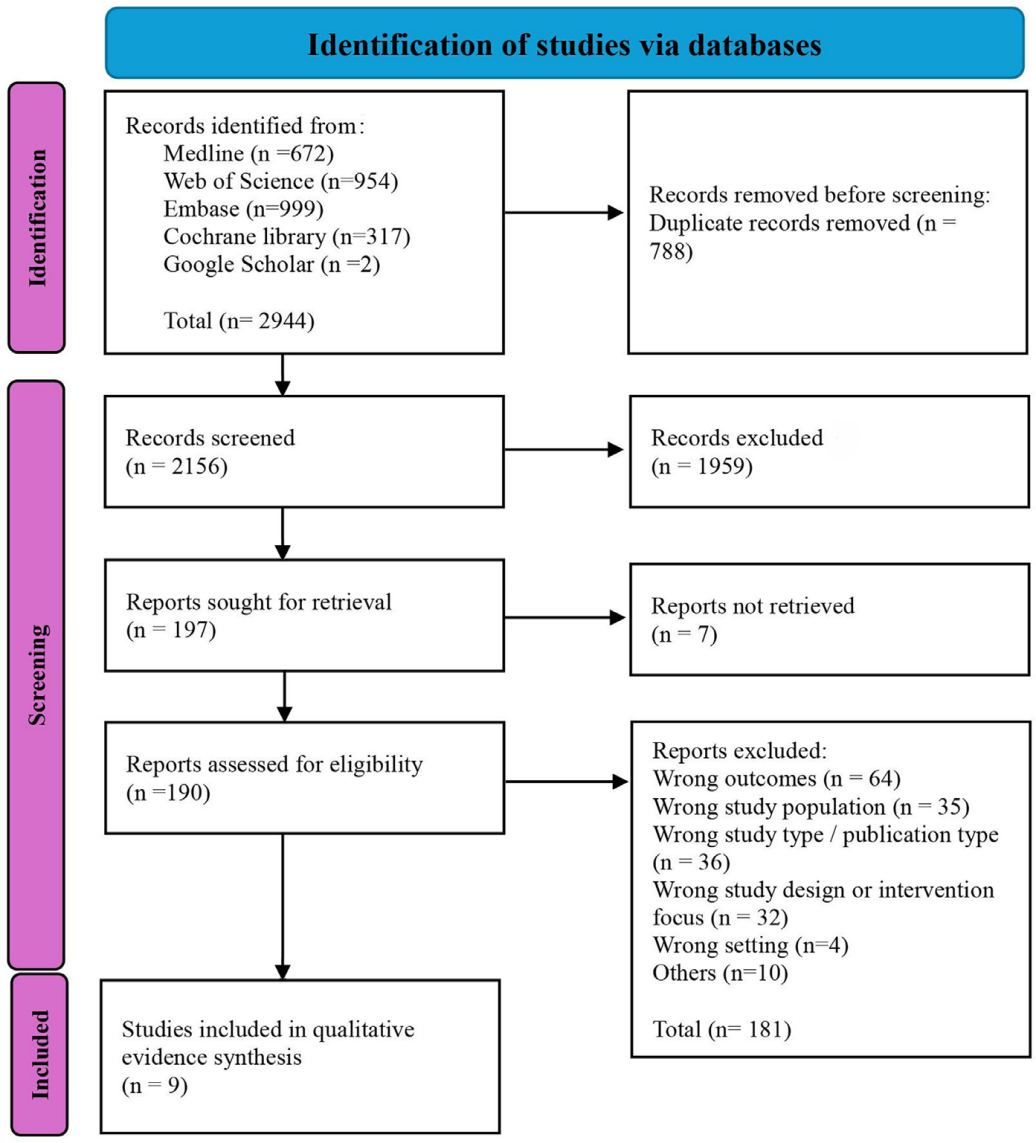
PRISMA flow diagram of study selection in the qualitative evidence synthesis.

The database searches resulted in 2944 articles, with 788 duplicates removed. The titles and abstracts of 2156 articles were screened, and 1959 were excluded according to the inclusion and exclusion criteria. The full texts of the remaining 197 articles were screened, and only nine studies met the criteria and were included in this synthesis. The details of the study selection process are presented in figure 1 (PRISMA flow chart).

### Description of included studies

Table 1 summarises the key characteristics of the six qualitative and three mixed-methods eligible studies. Six studies focused on CRC,^26^,^31^ two on cervical cancer^32 33^ and one on cancer screening in general.^34^ The studies were conducted in the USA (n=4), Canada (n=3), France (n=1) and Ghana (n=1). The sample sizes ranged from 12 to 29; data were collected using semi-structured interviews; and data was subjected to thematic synthesis (n=6), constant comparative analysis (n=1), grounded theory (n=1) and ‘interpretive’ analysis (n=1) (table 1).

**Table 1:** Characteristics of the included studies in qualitative evidence synthesis

No	Author (year)	Country	Aim	Study design	Sample size of primary care doctors	Data collection	Methodology	Data analysis
1	Guerra *et al* (2007)^26^	USA	To explore the barriers of and facilitators to physician recommendation of CRCS	Qualitative study	29	In-depth interviews, semi-structured interviews, focused group discussions	Qualitative study	Grounded theory
2	Lockman *et al* (2024)^34^	USA	To understand how healthcare providers perceive systemic- and individual-level factors affecting rural patients’ access to care and how this information impacts providers’ approach to cancer screening and prevention recommendations	Qualitative study	–	Semi-structured interviews	Qualitative study	Thematic analysis
3	Levy *et al* (2007)^29^	USA	To describe physicians’ reasons for screening or not screening specific patients for CRC and their approach to CRC testing discussions.	Mixed methods study	15	Telephone interview	Mixed methods study	Thematic analysis
4	Perkins *et al* (2024)^33^	USA	To explore factors associated with clinician-reported guideline-concordant screening, as well as facilitators and barriers to appropriate cervical cancer screening	Mixed methods study	12	Qualitative interviews by video conference	Mixed methods study	Thematic content analysis
5	Bapuji *et al* (2012)^27^	Canada	To explore physician beliefs about faecal occult blood testing (FOBT) and strategies they employed to enhance patient adherence	Qualitative study	15	Semi-structured interviews	Qualitative study	Interpretive description (ID)
6	Buchman *et al* (2016)^28^	Canada	To investigate overall CRC screening rates, patterns in the use of types of CRC screening, and sociodemographic characteristics associated with CRC screening and to gain insight into physicians’ perceptions about and use of FOBT or colonoscopy for patients at average risk of CRC.	Mixed methods study	29	Semi-structured telephone interviews	Mixed methods study	Thematic analysis
7	Lobchuk *et al* (2012)^30^	Canada	To address the gap in understanding the perspectives of primary care physicians, individuals at average-risk for colorectal cancer, and family on family role in promoting adherence to FOBT screening.	Qualitative study	15	Semi-structured interviews	Qualitative study	Content analysis and constant comparative techniques
8	Mignot *et al* (2024)^32^	France	To understand what leads to the non-adherence to the cervical cancer screening (CCS) recommendations during a consultation.	Qualitative study	24	In-depth semi-structured interviews	Qualitative study	Thematic analysis
9	Lussiez *et al* (2022)^31^	Ghana	To better understand the factors driving screening adherence and perceived barriers identified in an earlier quantitative study	Qualitative study	14	Semi-structured interviews	Qualitative study	Inductive thematic analysis

### Assessment of the quality of included studies

Using the CochrAne qualitative MEthodological LimitatiOns Tool (CAMLOT),^24^ most articles posed only minimal concern^2629^,^31^ or minor concern^2832^,^34^; and the remaining article raised moderate concern^27^ - see online supplemental appendix A, table 2.

### Qualitative review findings

The synthesis of qualitative data from individual studies comprised three salient themes, and details of codes, sub-themes and themes are presented in table 2.

**Table 2:** Theme development regarding PCD’s adherence to cancer screening guidelines

Code	Sub-theme	Descriptive theme	Analytical theme
Believe in guideline effectiveness	1.1. Factors influencing decision-making about screening	Views about utilisation of cancer screening guidelines	Views about cancer screening guidelines and screening decision making
Complications of colonoscopy			
End of screening			
False negative FOBT tests			
Doctors’ experience and recommendation			
Patient gender			
Patient age			
Anxious patients			
Patients with previous history of cancer			
Patients with symptoms			
Stable patient			
Patient acceptance for screening			
Patient request for screening			
Patients with family history of cancer			
Attend clinics together with family			
Family member encouragement and support			
Family members as role models for screening			
Prefer way of screening	1.2. Perceptions and preferences for screening tests		
Doctor being routine screener			
Doctor experience with family member or patient with cancer			
Teaching residents			
Positive attitudes towards screening			
Reviewing patient chart			
Patient history of refusal	2.1. Challenges related to delivery of screening	Barriers for primary care providers to perform cancer screening	Challenges in implementing cancer screening in primary care
Comorbidities			
Fear of colonoscopy (screening)			
Lack of health insurance			
Patients lack of interest for screening			
Patients’ belief that screening is only for symptomatic			
Present with other illness			
Uncomfortable or embarrassment for screening			
Patient did not have screening before			
Non-compliance of patients			
Patients do not trust doctors			
Assumption based screening			
Fatigue	2.2. PCD-related factors		
Frustration			
Forgetfulness			
Time constraints of doctors			
Doctors’ attitudes towards involvement of family			
Limited knowledge of doctors on screening tests			
Language barrier with patients			
Patients having another visit			
Discrepancies in medical advice			
Doctor’s strategies in screening recommendation			
Concurrent care with specialist or at hospital			
Acute care visit	2.3. Challenges related to health services and resources		
Availability of test kits			
Delay for confirmatory test			
Lack of facility for colonoscopy			
Lack of reminder for doctors			
Lack of screening record in system			
Confusing guidelines			
Discrepancy in screening recommendation period			
Need for uniform instruction to patients			
Stigma on cancer diagnosis	2.4. Challenges related to cancer awareness and beliefs		
Beliefs in traditional medicine			
Religious beliefs of patients			
Apps for screening and follow-up	3.1. Facilitating factors related to health services and resources	Facilitators for primary care providers to perform cancer screening	Factors enhancing cancer screening practices in primary care
Patient reminders system			
Networking and screening data record			
Opportunistic screening			
Data record			
Evidence-based screening guidelines			
Community awareness on screening	3.2. Facilitating factors related to community awareness		
Public education campaign			
Involving religious leaders for patient education			

### Theme 1. Views about cancer screening guidelines and screening decision making

#### Sub-theme 1.1. Factors influencing decision-making about screening

PCDs expressed positive views and reported that they followed cancer screening guidelines.^33^ *“I’m just going based off of the CDC, and AAFP, the American Association of Family Physicians, since I’m a family practitioner. I’m kind of going by what they are saying. I mean, if that’s what the results show, if that’s what the testing shows, that the benefit vs the risk ratio is better with a 3 year screen”* (USA).^33^ Junior PCDs faced challenges initially, but over time, they gained confidence in decision-making about recommending cancer screening tests. *“…I had a hard time at the beginning, when I was a young doctor, it wasn’t easy but after a while things started to come together. …”* (France).^32^ PCDs also reported negative views, particularly about inaccurate FOBT results and colonoscopy complications^27 28^: “*we have cases where negative tests and patients show up with cancer”* (Canada)^27^; and “*I’ve had 2 patients who’ve had bleeds from colonoscopies*” (Canada).^28^ Aside from these challenges, PCDs noted that they considered several patient factors like age, gender, medical and psychosocial status when making recommendations.^26 29 32 33^ It was recognised that patients with a personal history of cancer^26 29^ or who had had cancer symptoms were motivated strongly to follow through with screening recommendations *“(She) had had breast cancer times two. I advised her there was an association between breast cancer and colon cancer and this was the motivation for her to move forward with the colonoscopy*.” (USA).^29^ Patient-initiated requests for cancer screening were reported to lead to compliance with recommendations.^26^ Finally, it was noted that family support, prior family screening experience, and cancer family history^26 29 30^ increased adherence by doctors to the application of screening guidelines: “*Well, obviously if you have a partner who’s nagging the other one that would be helpful in increasing compliance rates*.” (Canada).^30^

#### Sub-theme 1.2. Perceptions and preferences for screening tests

PCDs’ preference for screening tests were influenced by resource availability, test accuracy, and system constraints.^27^,^29^ FOBT was viewed as an effective test,^27^ but some frustration was expressed over lack of ‘compensation’, “*I get nothing … they just want faecal occult blood testing and I kind of find that backwards…Yet I’m screening and not getting any compensation”* (Canada).^28^ PCDs preferred colonoscopy because of its ability to detect pathology beyond the reach of sigmoidoscopy and longer screening intervals.^29^ PCDs appeared to try to adhere consistently to guidelines for CRC screening.^26^ Personal exposure (via a family member or friend) or clinical exposure to cancer added to the motivation to undertake consistent screening practice; *“I mean it is just a matter of being obsessive or paranoid or being a good doctor or whatever you want to call it. It is sweating the details. You know that is what makes me do what I do is the desire to do the right thing.” (USA*).^26^

### Theme 2. Challenges in implementing cancer screening in primary care

#### Sub-theme 2.1. Challenges related to delivery of screening

PCDs reported several patient-related barriers to cancer screening. A patient history of refusal to screen hindered subsequent efforts to achieve screening uptake^26 29^: “*he has not been screened because he refused to undergo any type of screening*” (USA).^29^ Other barriers were presence of comorbidities, patients’ fears of procedures and financial constraints, particularly lack of insurance.^26 29^ “*I* (a PCD) *do look at the financial aspect of it and the insurance….I know their diabetes is out of control, do I spend the money on the diabetic medication or on the screening colonoscopy?*” (USA).^34^ Some patients declined to undergo CRC screening because they viewed screening as necessary only when symptoms appeared even after screening was discussed and explained by their doctor,“…[a patient was not]…*having a colonoscopy done unless he was having some symptoms that would require it*” (USA).^29^ The main reason why a patient visits primary care, and whether they are alone or with family members may make it hard or inappropriate for a PCD to discuss and recommend screening, *“When women come here, they come for shoulder pain, for the kid’s prescription. To say, the gynaecological examination will be done in another consultation, is difficult”* (France).^32^ Overall, PCDs from low- and middle-income countries (LMICs) like Ghana appeared to face greater difficulty due to religious and cultural beliefs held by patients^31^ than PCDs in high-income countries (HICs) such as the USA and France.^29 32^

#### Sub-theme 2.2. PCD-related factors

PCD-related factors such as fatigue,^26^ frustration^26^ and forgetfulness^26 29^ and time constraints^29^ led to missed opportunities to recommend screening: *“I think the reason that he wasn’t screened, and probably several other patients, was just a matter of time in not getting everything done”*.^29^ Limited knowledge about guidelines^33^ and the language barrier with non-English speaking patients further complicated the process.^26^ The presence of a translator took up consultation time to the extent that PCDs were unable to address preventive care.^26^ PCDs also assumed in certain occasions that cancer screening had been undertaken by other providers when patients were under concurrent care^26 29^:*“He gets his physicals at the VA hospital, so I really haven’t considered myself in the position to offer him screening …”*.^29^

#### Sub-theme 2.3. Challenges related to health services and resources

The mode of delivery of healthcare may make PCDs hesitate to recommend cancer screening. For example, inconsistent instructions to patients from different sources left patients confused about screening.^27^ *“The lab gives them* (patients) *written instructions to do opposite to what I* (PCD) *say. Need to have uniform instructions…“* (Canada).^27^ Resource limitations such as lack of FOBT tests may hinder implementation of screening recommendations^31^: *“If these tests are not available…. they will lose interest in it.”* (Ghana).^31^ Waiting lists and delays were another challenge in adhering to cancer screening.^26 27^ “*Getting colonoscopy done here* (Canada) *is difficult. Waiting times right now are 6 months to a couple of years*”.^27^ Additionally, lack of a reminder system and limited linkage in an electronic record may contribute to missed opportunities for tests^26 34^: *“we try to make sure our pap smear screens are up to date, and getting that into the electronic medical record has been difficult at times…so the percentage of our screening is lower than the reports that we’re getting from patients.”* [USA].^34^

#### Sub-theme 2.4. Challenges related to cancer awareness and beliefs

Some PCDs reported that cancer-related stigma was a major barrier to screening: *“cancer in itself is terrible. The stigma and also the unavailability of oncology facilities…so yeah, it’s a terrible thing to be diagnosed*.” (Ghana).^35^ Limited awareness about cancer, cultural norms, family beliefs and habits in rural communities may prevent acceptance and utilisation of preventive cancer screening (USA).^34^ Moreover, cultural beliefs and patient beliefs appeared to play a role in cancer screening recommendations: “(some patients)*…perceive cancer as a strange illness, they attend to it at home or in churches but not in the hospitals*” (Ghana).^35^

### Theme 3. Factors enhancing cancer screening practices in primary care

#### Sub-theme 3.1. Facilitating factors related to health services and resources

Integration of digital tools and reminder systems to engage and monitor patients were viewed by PCDs as facilitating factors for implementing cancer screening recommendations.^26 33^ *“Within EPIC* [electronic medical record provider system]*, it automatically gave you the option to select if you’re doing Pap only or Pap with Reflex HPV vs* (USA).^33^ Networking and screening data recording also appeared to motivate PCDs to implement guidelines, “*I am definitely promoting it more, because of the Physician Integrated Network (PIN6). I think that helps motivate because they [PIN] are capturing [FOBT] data*” (Canada).^27^

#### Sub-theme 3.2. Facilitating factors related to community awareness

PCDs stressed the need for public health education on cancer awareness. *“The Ministry of Health needs to really invest time and create more public awareness…public education is very necessary”* (Ghana).^31^ There is a need to include religious and community leaders in the dissemination of cancer literacy and in efforts to improve trust within local communities^31^: *“clinicians for instance…help educate religious leaders use their church platforms and mosque and religious setting to preach and to educate the people so that people will come our way” (Ghana*).^31^

### Confidence in qualitative review findings

Overall, CERQual confidence in review findings was either high (n=5 sub-themes) or moderate (n=3 sub-themes). The CERQual-rated inadequacy of qualitative data and limited geographic context contributed to the downgrading of the confidence level for some studies. Regarding the coherence of the findings regarding the subthemes, there were no concerns or minor/very minor concerns. No concerns or only very minor concerns were found regarding the criterion about the relevancy of sub-themes/findings in relation to the review question. Details about the CERQual’s components are presented in online supplemental appendix A, table 3.

The details of ENTREQ checklist could be seen in online supplemental appendix B.

## Discussion

This review searched systematically for qualitative studies on PCDs’ views about following screening guidelines and recommending breast, colorectal and cervical cancer screening. The qualitative evidence from the individual studies was synthesised and, overall, found that the medical practice of cancer screening in primary care was influenced by multiple factors including the perceptions and previous experience of PCDs, patient factors, the healthcare system and cultural norms.^26^,^3032 35^ In contrast to the systematic review which reported variation in adherence to cancer screening guidelines in primary care,^36^ this review did not find evidence for significant variation although that the synthesis focused exclusively on qualitative studies and did not explicitly investigate adherence variation. Furthermore, the synthesis appeared to indicate a consistency among PCDs across themes and subthemes; and the results pointed to clear motivation to follow national guidelines for cancer screening even when faced with various implementation challenges. However, several knowledge gaps were uncovered.

A US study including PCDs^34^ found that patient perspective, priorities, values, culture and financial level influenced the practice of recommending cancer screening. Similarly, a constellation of factors including limited availability of resources, healthcare system constraints and personal factors were reported in other studies to affect a PCD’s adherence to cancer screening recommendations.^29^,^3335^ However, the impact of these commonly reported challenges on adherence to screening guidelines was not quantified but appeared to be minimal according to this evidence synthesis. Collectively, the findings from the nine qualitative studies showed that PCDs trusted evidence-based guidelines, but implementation in terms of adherence and application varied with clinical experience and complications such as false iFOBT results and bleeding from colonoscopy, which affected the consistency of adherence.^28 32 33^ For example, junior doctors struggled initially to follow guidelines, but adherence improved over time with experience.^32^ There appeared to be a need to deliver targeted training for doctors, nurses and lab staff in order to improve patient information about screening and the consistency of communication relayed by and to different professionals.

PCD-related factors such as fatigue, frustration, forgetfulness and time constraints^26 29 30^ alongside limited knowledge, inaccurate risk assessments^33^ and language barriers^26^ caused missed screening opportunities; and there is a need to ensure a coordinated approach to cancer screening in situations where patients attend multiple healthcare professionals and to avoid a PCD assuming that screening was undertaken elsewhere.^26 29^ It is likely that addressing PCD workload and collaboration between primary care and specialists and healthcare professionals will increase adherence to cancer screening guidelines. Patient-related factors included fear, refusal and misconceptions that screening was only for symptomatic individuals^26 29^; and personal factors, like comorbidities, culture and religion appeared to impact adherence.^26 32 35^ Moreover, systematic issues like limited resources and delays in screening may hinder cervical cancer screening.^37^ Addressing healthcare service-related barriers and resource availability is likely to help PCDs to recommend and undertake cancer screening as well as avoiding delays in the scheduling of tests^26 27^ and providing a reminder system.

A qualitative exploration among PCDs in Australia showed that the lack of incentives for screening, challenges in screening data records and reminder systems, influenced their cancer screening programme.^38^ Moreover, practical challenges, such as limited resources and the unavailability of data storage systems, and reminder systems, are more apparent in LMI countries.^39^ Availability of cancer screening kits, such as FOBT, increases the likelihood of PCDs’ recommendation and highlights the need for kits, referral pathways and the timely provision of recommended screening. A further challenge to implementing cancer screening revolves around the need to change community and social norms^34^ relating to cancer screening^35^ in order to overcome the reluctance and hesitation in, for example, some communities due to patients’ fears and stigma about cancer.^31^ This reluctance is rooted to a significant degree in culture and, so, a culturally sensitive approach is important. Many of these barriers and facilitators have been reported previously. In addition to confirmation, this review provides an integrated synthesis and overview understanding about a PCD’s decision-making process. Our synthesis found that clear guidelines help PCDs make consistent recommendations,^26 29^ and PCDs’ beliefs and confidence in guidelines were reflected in their adherence to guidelines.^26 29 33^ Targeted educational workshops and training programmes may improve adherence.^40^ There may be scope for increasing community awareness, developing culturally sensitive and faith-based interventions and involving religious leaders with the aim of improving patient understanding and uptake of cancer screening.^26 31^ Most included studies were conducted in high-income countries, indicating a gap in qualitative evidence from low- and middle-income countries. In addition, qualitative studies exploring primary care doctors’ perceptions of breast cancer screening remain limited, highlighting the need for further research in this area.

### Strengths and limitations

This QES explored PCDs’ perceptions and experiences in relation to recommending and implementing cancer screening guidelines. Although studies were searched without geographical limits, most studies were from HICs (USA, Canada, France), with only one from a LMIC (Ghana). Thus, findings may reflect PCDs in HICs. While the review addressed three cancer site screenings, most studies focused on CRC and cervical cancer. Only nine articles were included in this review indicating the limited availability of qualitative studies exploring PCD’s perspective about the delivery of cancer screening. A further limitation relates to the use of English language only in the search and selection of studies. Moreover, the papers in the review lacked detailed description of contextual factors such as national or regional health system characteristics, funding models and the presence of organised screening programmes. Health system structures and screening recommendation guidelines may have changed over time since the publication of papers in the review. Therefore, the transferability of findings to different healthcare systems and settings should be interpreted with this potential limitation in mind.

## Conclusion

Overall, PCDs across various countries and healthcare systems generally followed screening guidelines for CRC and cervical cancer and there is a need for qualitative studies about PCDs adherence to breast cancer guidelines. The qualitative evidence synthesis indicated that a range of factors may facilitate or inhibit adherence by PCDs to following recommended screening guidelines. Further qualitative studies are required in LMICs to explore cancer screening in resource-constrained primary care settings.

## Supplementary material

10.1136/bmjopen-2025-109989 online supplemental file 1

10.1136/bmjopen-2025-109989 online supplemental file 2

10.1136/bmjopen-2025-109989 online supplemental file 3

## Data Availability

Data are available upon reasonable request.
