# Supplementary material for: What influences the decision by primary care doctors to recommend cancer screening? A qualitative evidence synthesis
Source: BMJ Open. 2026 May 24;16(5):e109989. doi: 10.1136/bmjopen-2025-109989 (PMC13202075; doi:10.1136/bmjopen-2025-109989)
Supplement: online supplemental file 1 [file bmjopen-16-5-s001.pdf]

Appendix A:

Supplemental Table 1. Search terms relating to adherence by primary care doctors to cancer screening guidelines

Concept	Search terms
Concept 1	primary healthcare doctor* OR primary healthcare physician* OR doctor* OR physician* OR general practitioner* OR GP*<br>AND
Concept 2	colorectal cancer OR breast cancer OR cervical cancer<br>AND
Concept 3	screening guideline* OR guideline* OR practice guideline* OR clinical practice guideline*<br>AND
Concept 4	Adherence OR compliance OR practice pattern OR utilization, non-adherence, non-compliance

Supplemental Table 2: Quality assessment of the included studies

ID	Author (Year)	Meta Domain				Method Domains- Research Design				Method Domains - Research Conduct				
		Aim & Questions	Stakeholders	Researchers	Context	Research Strategy	Theory	Ethical Consideration	Equity, Diversity, and Inclusion	Participant Recruitment & Selection	Data Collection	Analysis & Interpretation	Presentation of Findings	Overall
1	Guerra, CE, et al (2007)	E	UC	G	F	E	G	G	G	G	G	G	G	Minimal
2	Bapuji SB, et al. (2012)	E	UC	G	G	E	G	P	G	G	G	G	G	Moderate
3	Buchman S, et al. (2016)	E	UC	G	G	E	F	G	F	G	G	G	G	Minor
4	Levy BT, et al. (2007)	E	G	G	G	E	UC	G	F	G	E	G	G	Minimal
5	Lobchuk MM, et al. (2012)	E	UC	G	F	E	G	G	G	G	G	G	G	Minimal
6	Lussiez A, et al. (2022)	E	UC	G	G	E	G	G	G	F	G	G	G	Minimal
7	Lockman, et al. (2024)	E	UC	G	F	E	F	G	E	F	G	G	G	Minor
8	Mignot, et al. (2024)	E	UC	G	G	E	F	G	E	F	G	G	G	Minor
9	Perkins, et al. (2024)	E	UC	G	G	E	UC	G	G	G	G	G	G	Minor

Quality assessment

E = Excellent, G = Good, F = Fair, P = Poor, UC = Unclear

Supplemental Table 3: GRADE-CerQual Summary of Qualitative Evidence Synthesis Results

	Findings	Methodological limitations	Coherence	Adequacy	Relevance	Overall confidence	Contributing records
**THEME 1. Perception on Cancer Screening Guidelines and Screening Decision Making**							
1	Subtheme 1.1. “Factors influencing decision-making about screening "<br><br>Trust in evidence-based cancer screening guidelines and adherence to screening were reported in some primary care providers (PCPs). Their confidence in cancer screening recommendation and communication developed with working experience. Screening recommendations were also influenced by patient-related factors, including age, gender, health awareness, and acceptance. Family involvement, through support and encouragement, further facilitated screening uptake.	Minor concerns	Minor concerns	No/Very minor concerns	No/Very minor concerns	High confidence	Bapuji et al. 2012; Buchman et al. 2016; Guerra et al. 2007; Levy et al. 2007; Mignot et al. 2024; Perkins et al. 2024
2	Sub-theme. 1.2. "Perceptions and preferences for screening tests"<br><br>Primary healthcare providers’ preference on screening measures was influenced by availability of resources, accuracy of screening measures, and healthcare system limitations. Reviewing patient records, personal, and clinical information supported their decision-making in recommending appropriate screening.	Minor concerns regarding	Minor concerns	Minor concerns	No/Very minor concerns	High confidence	Bapuji et al. 2012; Buchman et al. 2016; Guerra et al. 2007; Levy et al. 2007; Perkins et al. 2024

	Findings	Methodological limitations	Coherence	Adequacy	Relevance	Overall confidence	Contributing records
**THEME 2: Challenges in Implementing Cancer Screening in Primary Care**							
3	Sub-theme. 2.1.: "Challenges related to delivery of screening"<br><br>Patients' lack of interest in cancer screening, previous history of screening refusal could hinder PCPs to recommend cancer screening. PCPs tried to prioritize the patients' need, financial matter, availability of insurance when considering for cancer screening recommendation. Patient's misconception about cancer screening, cultural barriers, fear and embarrassment, and the primary healthcare setting also influence screening uptake.	Minor concerns	Minor concerns	Minor concerns	No/Very minor concerns	High confidence	Guerra et al. 2007; Levy et al. 2007; Lockman et al. 2024; Lussiez et al. 2022; Mignot et al. 2024
4	Sub-theme. 2.2.: "PCD-related factors"<br><br>Primary care providers' fatigue, frustration, and forgetfulness contributed to missed opportunities to discuss cancer screening with patients. Some PCP might also face challenges to screening due to limited knowledge about updated guidelines, personal judgement for screening, and inappropriate risk assessments. Which might cause barriers to making appropriate recommendations. Sometimes, concurrent healthcare with the specialists could lead to overlooked to screen the patients in primary care clinics.	Minor concerns	Minor concerns	Minor concerns	No/Very minor concerns	High confidence	Guerra et al. 2007; Levy et al. 2007; Lobchuk et al. 2012; Perkins et al. 2024
5	Sub-theme. 2.3. "Challenges related to health services and resources"<br><br>The nature of outpatient care may contribute for the less prioritization of colorectal cancer screening. Furthermore, delay performing colonoscopy to patients may further hinder the cancer screening process. Lack of screening reminder system, record	Minor concerns	Minor concerns	Minor concerns	Minor concerns	High confidence	Bapuji et al. 2012; Guerra et al. 2007; Levy et al. 2007;

	Findings	Methodological limitations	Coherence	Adequacy	Relevance	Overall confidence	Contributing records
	linage system in EMR further hinder to access the complete history of patient's cancer screening.						Lockman et al. 2024
6	Sub-theme. 2.4. "Challenges related to cancer awareness and beliefs"<br><br>Community-related barriers such as stigma for cancer diagnosis, fear, limited awareness, family beliefs about cancer screening hinder screening recommendation.	No/Very minor concerns	No/Very minor concerns	Moderate concerns	No/Very minor concerns	Moderate confidence	Lockman et al. 2024; Lussiez et al. 2022
**THEME 3: Factors Enhancing Cancer Screening Practices in Primary Care**							
7	Sub-theme. 3.1. "Facilitating factors related to health services and resources"<br><br>Ensuring the availability and accessibility of cancer screening test kits influence PCPs to recommend screening to their patients. In the digital era, integrating digital tools, reminder systems, and electronic health records enhances PCPs' ability to engage with patients and monitor cancer screening records.	Minor concerns	No/Very minor concerns	Moderate concerns	No/Very minor concerns	Moderate confidence	Bapuji et al. 2012; Guerra et al. 2007; Perkins et al. 2024
8	Sub-theme. 3.2. " Facilitating factors related to community awareness"<br><br>Raising awareness of cancer screening in community will improve the patients screening update and a facilitating the healthcare providers to recommend the cancer screening. Primary care providers also considered that they could educate the religious leaders, who will in turn educate the people in their respective religious community. This approach could facilitate to improve trust, acceptance, and encourage people for screening uptake.	No/Very minor concerns	No/Very minor concerns	Moderate concerns	No/Very minor concerns	Moderate confidence	Guerra et al. 2007; Lussiez et al. 2022
