# Supplementary material for: What influences the decision by primary care doctors to recommend cancer screening? A qualitative evidence synthesis
Source: BMJ Open. 2026 May 24;16(5):e109989. doi: 10.1136/bmjopen-2025-109989 (PMC13202075; doi:10.1136/bmjopen-2025-109989)
Supplement: online supplemental file 3 [file bmjopen-16-5-s003.pdf]

Appendix C

Search strategies

Search date: 22 January 2025

Medline

1. (primary healthcare doctor* OR primary healthcare physician* OR doctor* OR physician* OR general practitioner* OR GP*).mp.[mp=title, book title, abstract, original title, name of substance word, subject heading word, floating sub-heading word, keyword heading word, organism supplementary concept word, protocol supplementary concept word, rare disease supplementary concept word, unique identifier, synonyms, population supplementary concept word, anatomy supplementary concept word]

2. limit 1 to yr=”2000-current”

3. (colorectal cancer OR breast cancer OR cervical cancer).mp.[mp=title, book title, abstract, original title, name of substance word, subject heading word, floating sub-heading word, keyword heading word, organism supplementary concept word, protocol supplementary concept word, rare disease supplementary concept word, unique identifier, synonyms, population supplementary concept word, anatomy supplementary concept word]

4. limit 3 to yr=”2000-current”

5. (screening guideline* OR guideline* OR practice guideline* OR clinical practice guideline*).mp.[mp=title, book title, abstract, original title, name of substance word, subject heading word, floating sub-heading word, keyword heading word, organism supplementary concept word, protocol supplementary concept word, rare disease supplementary concept word, unique identifier, synonyms, population supplementary concept word, anatomy supplementary concept word]

6. limit 5 to yr=”2000-current”

7. (adherence OR compliance OR practice pattern OR utilization OR non-adherence OR non-compliance).mp.[mp=title, book title, abstract, original title, name of substance word, subject heading word, floating sub-heading word, keyword heading word, organism supplementary concept word, protocol supplementary concept word, rare disease supplementary concept word,

unique identifier, synonyms, population supplementary concept word, anatomy supplementary concept word]

8. limit 7 to yr=”2000-current”

9. 2 and 4 and 6 and 8

Web of science

(((ALL=(primary healthcare doctor OR primary healthcare physician OR doctor OR physician OR general practitioner OR GP))

AND

ALL=(colorectal cancer OR breast cancer OR cervical cancer))

AND

ALL=(screening guideline OR guideline OR practice guideline OR clinical practice guideline))

AND

ALL=(Adherence OR compliance OR practice pattern OR utilization OR non-adherence OR non-compliance)

Timespan: 2000-01-10 to 2025-01-22 (Publication Date)

Embase

('primary healthcare doctor*' OR 'primary healthcare physician*' OR 'doctor*' OR 'physician*' OR 'general practitioner*' OR 'GP*')

AND

('colorectal cancer' OR 'breast cancer' OR 'cervical cancer')

AND

('screening guideline*' OR 'guideline*' OR 'practice guideline*' OR 'clinical practice guideline*')

AND

('adherence' OR 'compliance' OR 'practice pattern' OR 'utilization' OR 'non-adherence' OR 'non-compliance')

Limit yr=”2000-current”

Cochrane Library

#1 primary healthcare doctor* OR primary healthcare physician* OR doctor* OR physician* OR general practitioner* OR GP*

2 colorectal cancer OR breast cancer OR cervical cancer

#3 screening guideline* OR guideline* OR practice guideline* OR clinical practice guideline*

#4 adherence OR compliance OR practice pattern OR utilization OR non-adherence OR non-compliance

#1 AND #2 AND #3 AND #4

with Cochrane Library publication date from Jan 2000 to Jan 2025
