# Supplementary material for: What influences the decision by primary care doctors to recommend cancer screening? A qualitative evidence synthesis
Source: BMJ Open. 2026 May 24;16(5):e109989. doi: 10.1136/bmjopen-2025-109989 (PMC13202075; doi:10.1136/bmjopen-2025-109989)
Supplement: Supplementary file 4 [file bmjopen-2025-109989.reviewer_comments.pdf]

PEER REVIEW HISTORY

BMJ Open publishes all reviews undertaken for accepted manuscripts. Reviewers are asked to complete a checklist review form and are provided with free text boxes to elaborate on their assessment. These free text comments are reproduced below.

ARTICLE DETAILS

Title (Provisional)

What influences the decision by primary care doctors to recommend cancer screening? A qualitative evidence synthesis

Authors

Htay , Mila Nu Nu; Oo, Mon Mon Thawda; Su, Tin Tin; Donnelly, Michael

---

VERSION 1 - REVIEW

**Reviewer**	**1**
**Name**	**Cithambaram, Kumaresan**
**Affiliation**	**Technological University of the Shannon**
**Date**	**05-Oct-2025**
**COI**	**None**

Thank you for the opportunity to review this paper. The topic is timely and of significant importance to cancer prevention and early detection in primary care settings. The manuscript is generally well structured and demonstrates a clear effort to follow systematic review and qualitative evidence synthesis approach. However, several areas require clarification and refinement to strengthen the methodological transparency, coherence between sections, and overall presentation. My detailed comments below are intended to support the authors in improving the clarity, consistency, and rigour of the paper.

Title

Please reconsider the title to ensure it accurately represents the aim and content of the paper.

Abstract

Background: Please clearly outline the importance of primary care doctors (PCDs) in cancer screening and justify the need for a review focusing on PCDs' perceptions and experiences.

Methods: I suggest to remove "evidence synthesis findings" and replace it with "Review findings." Reword "thematic analysis" as "thematic synthesis" to align with the Thomas and Harden approach.

Results: The sentence "The analysis of the evidence produced a synthesis that comprised three main analytical themes" should be revised to "The synthesis of evidence identified three main analytical themes."

Correct the enumeration sequence from "(i)… (ii)… and (ii)" to "(i)… (ii)… and (iii)."

The phrase “at least relating to CRC and to a lesser extent, cervical cancer” is unclear; please clarify. Expand abbreviations (CRC, BC, LMIC) at first mention.

Include relevant keywords such as primary care doctors and qualitative evidence synthesis.

Strengths and Limitations: This section may be more appropriately placed at the end of the manuscript, following the discussion, unless the journal’s guidelines specify otherwise.

Introduction

The phrase “improved prevention” is vague; clarify whether it refers to lifestyle interventions, vaccination, or another preventive approach. “Screening is key to reducing the burden” is too generic—specify which burden (e.g., morbidity, mortality, healthcare costs).

Provide a reference for the statement: “Primary care doctors (PCDs) are expected to play a valuable role in cancer prevention and early detection as they are the first point-of-contact in most healthcare systems.”

Expand the discussion explaining why variations occur and what factors influence them—for instance, the cited statistic from Poland would benefit from contextual explanation.

Methods

Inclusion and Exclusion Criteria:

The search strategy suggests inclusion of screening guidelines; however, it is unclear whether national screening service websites or grey literature were searched. Please specify which websites or databases (if any) were included.

Reword “The search results were exported to COVIDENCE systematic review” to “The search results were exported to Covidence, a software platform for managing systematic review screening.”

Clarify the review process: “Each title and abstract was reviewed by two independent reviewers” — specify whether screening was conducted in pairs or if all four reviewers independently screened the records. Similarly, indicate which reviewers conducted the full-text screening.

Describe how the data extraction form was developed and piloted. The phrase “conclusion from results and conclusion sections” should be reworded for precision. Identify the two independent researchers who conducted data extraction.

Replace “Evidence synthesis” with “Data synthesis.” Indicate which reviewers were involved in theme development. Change “Thomas and Harden thematic analysis” to “Thomas and Harden’s thematic synthesis.”

Rephrase “Study findings were assessed for methodological limitations” to “The review findings were assessed for methodological limitations.” Provide a reference for the CERQual approach.

The section “Assessment of confidence in synthesis” needs to be rewritten. The CAMLOT tool assesses methodological limitations of included primary studies and should be described separately in the Methods section.

Reflexivity: While team composition is appropriately noted, the section would benefit from a more explicit description of how researchers' disciplinary and cultural backgrounds, particularly their emphasis on primary care and local perspectives on cancer screening, shaped coding and interpretation. Clarify what strategies were used to identify and mitigate bias (e.g., reflexive memoing, triangulation, and team debriefings).

State the reasons for exclusion of studies after full-text screening: "The full texts of the remaining 197 articles were screened, and only nine studies met the inclusion criteria."

Correct the sentence to: "Six studies focused on CRC [22–27], two on cervical cancer [28, 29], and one on cancer screening in general."

Ensure accuracy in study count: if nine studies were included, specify the country of origin of the ninth study (currently, only eight are accounted for: U.S. [n=3], Canada [n=3], France [n=1], Ghana [n=1]).

Review Findings

Replace "Qualitative evidence synthesis findings" with "Review Findings."

Ensure consistency between the abstract and results section: the abstract mentions three analytical themes, while the results state four salient themes.

Sub-theme 1.1 ("Experience in the use of guidelines and patient considerations") does not align with the supporting evidence, which focuses on factors influencing screening decisions rather than direct experiences using guidelines. Consider renaming or reorganising this sub-theme for conceptual alignment.

In Sub-theme 1.2, the claim that "PCDs appeared to try to adhere consistently to guidelines" lacks sufficient evidence; this should be substantiated or revised.

Reassess the structure and clarity of all sub-themes and ensure Sub-theme 2.4 is included in the abstract.

Replace "Confidence in evidence synthesis findings" with "Confidence in the review findings."

Discussion

The discussion section is strong, with appropriate engagement with the literature to support the review's findings.

General Comment

Ensure consistent use of British or American spelling throughout the manuscript (e.g., programme vs program, synthesised vs synthesized).

Please send the ENTREQ reporting guideline.

**Reviewer**	**2**
**Name**	**Sussman, Jonathan**
**Affiliation**	**McMaster University, Division of Radiation Oncology**
**Date**	**23-Feb-2026**
**COI**	**None**

This paper is an attempt to synthesize qualitative studies of enablers and barriers to screening breast, colorectal and cervical cancer from the perspectives of primary care physicians.

the authors describe a systematic screening system to identify publications of interest and from an initial screen of over 2500 papers find 9 that are included. I find this quite surprising as the topic area has been studied quite extensively and this sample of 9 papers with 12-29 participants per paper seems quite limited in terms of robustness and validity of findings.

A major limitation of the paper is the lack of any description of the screening context in the papers-national or regional health system, funding model (of system and provider), guidelines and if any screening programs exist. The barriers and enablers identified do not seem to be much different from those that have been described previously in the literature so I am unsure what these findings add as currently presented.

VERSION 1 - AUTHOR RESPONSE

Reviewer: 1

Comments to the Author:

Thank you for the opportunity to review this paper. The topic is timely and of significant importance to cancer prevention and early detection in primary care settings. The manuscript is generally well structured and demonstrates a clear effort to follow systematic review and qualitative evidence synthesis approach. However, several areas require clarification and refinement to strengthen the methodological transparency, coherence between sections, and overall presentation. My detailed comments below are intended to support the authors in improving the clarity, consistency, and rigour of the paper. Please send the ENTREQ reporting guideline.

Title

1. Please reconsider the title to ensure it accurately represents the aim and content of the paper.

Response: Thank you for the valuable suggestion. We have revised the title to include the research question and to reflect the research focus on factors influencing primary care doctor’s decision making to recommend for cancer screening.

“What influences the decision by primary care doctors to recommend cancer screening? A qualitative evidence synthesis”

Abstract

2. Background: Please clearly outline the importance of primary care doctors (PCDs) in cancer screening and justify the need for a review focusing on PCDs' perceptions and experiences.

Response: Thank you for the suggestion. We have outlined the importance of primary care doctors (PCDs) in cancer screening as first-contact providers in community as follows:

"PCDs tend to fulfil the role of 'gatekeeper' in their respective country's health system and in relation to this role, initiate cancer screening discussions and when appropriate, recommend screening tests [15]. Indeed, a doctor's recommendation is one of the strongest predictors of patient participation in cancer screening programmes [16, 17]." (Line: 95-99)

We have also strengthened the rationale for focusing specifically on PCDs' perceptions and experiences, highlighting the absence of prior qualitative evidence syntheses in this area as follow:

"Previous research including reviews that examined the factors influencing adherence to cancer screening guidelines, focused mainly on provider-patient communication patterns, and specific age groups [12, 17-19]. To the best of our knowledge, there is no qualitative evidence synthesis of primary care doctors' experiences and perceptions regarding their decision to recommend breast, cervical, and colorectal cancer screening across age groups. Thus, this ENTREQ-guided review synthesised studies about PCDs' views regarding their decisions to recommend breast, cervical, and colorectal cancer screening in order to derive insights into cancer screening in primary care, including the use of guidelines, and related implementation challenges [20]." (Line: 106-122)

3. Methods: I suggest to remove "evidence synthesis findings" and replace it with "Review findings." Reword "thematic analysis" as "thematic synthesis" to align with the Thomas and Harden approach.

Response: Duly noted and we have revised as "Review findings" in the manuscript. (Line: 190, 235, 361, 363)

We have corrected the words as "thematic synthesis". (Line: 227)

4. Results: The sentence "The analysis of the evidence produced a synthesis that comprised three main analytical themes" should be revised to "The synthesis of evidence identified three main analytical themes."

Response: We have revised according to the suggestion in the abstract (result section). (Line: 19-20)

5. Correct the enumeration sequence from "(i)… (ii)… and (ii)" to "(i)… (ii)… and (iii)." The phrase "at least relating to CRC and to a lesser extent, cervical cancer" is unclear; please clarify. Expand abbreviations (CRC, BC, LMIC) at first mention.

Response: Duly noted and we have corrected as (i)…. (ii)… and (iii). (Line: 24-25)

6. Include relevant keywords such as primary care doctors and qualitative evidence synthesis.

Response: We have revised the keywords according to the valuable suggestions.

"Cancer, Primary Care, Guideline Adherence, Decision Making, Primary Care Doctors, Qualitative Evidence Synthesis" (Line: 50-52)

7. Strengths and Limitations: This section may be more appropriately placed at the end of the manuscript, following the discussion, unless the journal's guidelines specify otherwise.

Response: We fully understood the guidance. We have included at the first section according to the journal's guidelines. We have also included the brief explanation at the end of the manuscript. (Line: 445-463)

Introduction

8. The phrase "improved prevention" is vague; clarify whether it refers to lifestyle interventions, vaccination, or another preventive approach. "Screening is key to reducing the burden" is too generic—specify which burden (e.g., morbidity, mortality, healthcare costs).

Response: Thank you for the helpful suggestion. The prevention measures, such as reducing carcinogen exposure through tobacco control and vaccination for human papillomaviruses (HPV), should be encouraged. Therefore, we have revised in the introduction as follows;

"Addressing this challenge requires improved prevention such as vaccination for human papillomaviruses (HPV), tobacco control, early detection and treatment, and palliative and survivorship care". (Line:82-84)

9. Provide a reference for the statement: "Primary care doctors (PCDs) are expected to play a valuable role in cancer prevention and early detection as they are the first point-of-contact in most healthcare systems."

Response: Duly noted and we have included the reference of the statement in the manuscript. (Line: 92-93)

10. Expand the discussion explaining why variations occur and what factors influence them—for instance, the cited statistic from Poland would benefit from contextual explanation.

Response: Thank you for the suggestion. We have included the additional explanation of potential reasons for variation in screening practices. The main obstacles faced by the doctors at primary care are due to lack of time and lack of financial resources for providing cancer prevention services. We have included in the manuscript as follows;

"For example, 12.5% and 60.6% of doctors in Poland 'routinely' or 'sometimes' informed patients about cancer screening and risks, whilst less than half discussed CRC (31%), cervical (41%), and breast (44%) screening [12]. This screening practice variation may reflect the challenges faced in primary care setting such as lack of time and lack of financial resources for providing cancer prevention services [12]." (Line: 102-106)

Methods

11. Inclusion and Exclusion Criteria:

The search strategy suggests inclusion of screening guidelines; however, it is unclear whether national screening service websites or grey literature were searched. Please specify which websites or databases (if any) were included.

Response: Our review focused on peer-reviewed qualitative studies or mixed-methods studies indexed in databased. Ministry of health websites of the respective countries, national screening programme websites and grey literature were not included in the search strategy. We have clarified about this information in the Method section of the manuscript. (Line: 75-77; 127-129)

12. Reword "The search results were exported to COVIDENCE systematic review" to "The search results were exported to Covidence, a software platform for managing systematic review screening."

Response: We have amended the sentence as suggested. (Line: 143-144)

13. Clarify the review process: "Each title and abstract was reviewed by two independent reviewers" — specify whether screening was conducted in pairs or if all four reviewers independently screened the records. Similarly, indicate which reviewers conducted the fulltext screening.

Response: Members of the review team (MNNH, MMTO) paired up and screened the articles independently. Each title, abstract, and full text was reviewed by two independent reviewers (MNNH, MMTO) and discrepancies were resolved through discussion by with a third reviewer (MD). Any disagreements were discussed and resolved by the review team (MNNH, MMTO, MD, TTS). (Line: 148-152)

14. Describe how the data extraction form was developed and piloted. The phrase "conclusion from results and conclusion sections" should be reworded for precision. Identify the two independent researchers who conducted data extraction.

Response: We have included the development and piloting of the data extraction from. The data extraction from was initially developed based on the review objectives and relevant study information (such as sample, study setting, details of conduct of study methodology). The form was piloted with two included studies and it was found to be appropriate and relevant to continue to use for the rest of the included studies. The name of researchers who conducted data extraction are included in the manuscript as follows;

"A data extraction form was created based on the review objectives, relevant study information and methodology. The data extraction form (in Excel sheet mode and Microsoft Word document) was piloted using the two eligible papers and then it was used for the remaining eligible papers. Data from the included papers about study characteristics, methodology, and summary of findings were extracted onto an Excel sheet; and data regarding themes, findings, direct quotations, relevant tables, figures, and conclusion from results and conclusion sections, were extracted onto a Microsoft Word document. Data extraction was conducted by two independent researchers (MNNH, MMTO)." (Line: 154-164)

15. Replace "Evidence synthesis" with "Data synthesis." Indicate which reviewers were involved in theme development. Change "Thomas and Harden thematic analysis" to "Thomas and Harden's thematic synthesis."

Response: We have revised the term (sub-heading) as Data synthesis. We have also edited as Thomas and Harden's thematic synthesis. (Line: 169)

16. Rephrase "Study findings were assessed for methodological limitations" to "The review findings were assessed for methodological limitations." Provide a reference for the CERQual approach.

Response: Duly noted and we have edited as "The review findings were assessed for methodological limitations". The reference of the CERQual approach has been added to the manuscript as well. (Line: 192)

17. The section "Assessment of confidence in synthesis" needs to be rewritten. The CAMLOT tool assesses methodological limitations of included primary studies and should be described separately in the Methods section.

Response: The description of the CAMLOT tool for methodological limitation assessment has been separated from the "Assessment of Confidence" CERQual assessment section as follows;

"The methodological strengths and limitations of eligible primary studies (n=9) were assessed using the CochrAne qualitative Methodological LimitatiOns Tool (CAMLOT) [25]. CAMLOT evaluates potential methodological concerns in qualitative studies. The overall concern level about the studies was classified as: "no," "minimal," "minor," "moderate," or "serious". Two researchers (MNNH, MMTO) independently conducted the assessment, and any discrepancies were resolved through discussion." (Line 180-186)

18. Reflexivity: While team composition is appropriately noted, the section would benefit from a more explicit description of how researchers' disciplinary and cultural backgrounds, particularly their emphasis on primary care and local perspectives on cancer screening, shaped coding and interpretation. Clarify what strategies were used to identify and mitigate bias (e.g., reflexive memoing, triangulation, and team debriefings).

Response: Thank you for the insightful suggestion. The reflexivity section has been expanded to explicitly describe about the team composition. The strategies such as team discussion, reflexive consideration of interpretation, and consensus building was applied during the data synthesis process. (Line: 200-211)

19. State the reasons for exclusion of studies after full-text screening: "The full texts of the remaining 197 articles were screened, and only nine studies met the inclusion criteria."

Response: The reasons for exclusion after full-text review were not involving primary care doctors (wrong study population), not qualitative design (wrong study design), or not addressing screening recommendations (wrong outcome), etc. We have included the details of exclusion reason in the figure (PRISMA Flowchart).

20. Correct the sentence to: "Six studies focused on CRC [22–27], two on cervical cancer [28, 29], and one on cancer screening in general."

Response: Duly noted and we have corrected the sentence in the manuscript. (Line: 224-225)

21. Ensure accuracy in study count: if nine studies were included, specify the country of origin of the ninth study (currently, only eight are accounted for: U.S. [n=3], Canada [n=3], France [n=1], Ghana [n=1]).

Response: Thank you for the correction guidance. We have checked and edited the country distribution in the manuscript as follows;

"The studies were conducted in the USA (n=4), Canada (n=3), France (n=1), and Ghana (n=1)." Line: (225-226)

Review Findings

21. Replace "Qualitative evidence synthesis findings" with "Review Findings."

Response: Duly noted and we have corrected in the manuscript. (Line: 235)

22. Ensure consistency between the abstract and results section: the abstract mentions three analytical themes, while the results state four salient themes.

Response: We have corrected in the result section as "three analytical themes" and ascertain the consistency of reviewing findings. (Line: 19-28)

23. Sub-theme 1.1 ("Experience in the use of guidelines and patient considerations") does not align with the supporting evidence, which focuses on factors influencing screening decisions rather than direct experiences using guidelines. Consider renaming or reorganising this subtheme for conceptual alignment.

Response: The main theme and sub-theme has been revised to better reflect the supporting evidence regarding factors influencing screening decision making.

"Theme 1. Views about Cancer Screening Guidelines and Screening Decision Making

Sub-theme 1.1. Factors influencing decision-making about screening" (Line: 241)

24. In Sub-theme 1.2, the claim that "PCDs appeared to try to adhere consistently to guidelines" lacks sufficient evidence; this should be substantiated or revised.

Response: Duly noted and thank you for the concern for sub-theme 1.2. We have included the evidence from the study (Line: 277)

25. Reassess the structure and clarity of all sub-themes and ensure Sub-theme 2.4 is included in the abstract.

Response: We have the structure and included the sub-theme 2.4 in the abstract. (Line: 25-26)

26. Replace “Confidence in evidence synthesis findings” with “Confidence in the review findings.”

Response: Duly noted and we have revised as “Confidence in qualitative review findings”. (Line: 361)

Discussion

27. The discussion section is strong, with appropriate engagement with the literature to support the review’s findings.

Response: Thank you for kind and positive feedback for the discussion section.

General Comment

28. Ensure consistent use of British or American spelling throughout the manuscript (e.g., programme vs program, synthesised vs synthesized).

Response: We have carefully checked and revise the manuscript to ensure consistent use of British English (programme, synthesised).

The manuscript has been carefully checked and revised to ensure consistent use of British English spelling throughout the manuscript.

29. Please send the ENTREQ reporting guideline.

Response: We have included the ENTREQ checklist for the reporting of synthesis of qualitative research.

Reviewer: 2
Comments to the Author:
1. This paper is an attempt to synthesize qualitative studies of enablers and barriers to screening breast, colorectal and cervical cancer from the perspectives of primary care physicians.
the authors describe a systematic screening system to identify publications of interest and from an initial screen of over 2500 papers find 9 that are included. I find this quite surprising as the topic area has been studied quite extensively and this sample of 9 papers with 12-29 participants per paper seems quite limited in terms of robustness and validity of findings.

Response: Thank you for the thoughtful comment. While the cancer screening topic is widely studied across the countries, most of the scientific publications were quantitative studies. While the qualitative studies exploring physician’s perspective is

limited. Our review specifically aimed to synthesise qualitative studies exploring PCDs' perceptions and experiences regarding cancer screening recommendations. After applying these inclusion criteria, only nine studies met eligibility requirements. This limitation for generalisability have been acknowledged in the limitation section of the manuscript. (Line: 445-463)

2. A major limitation of the paper is the lack of any description of the screening context in the papers-national or regional health system, funding model (of system and provider), guidelines and if any screening programs exist.

Response: Thank you for this important comment. We acknowledge that our review does not provide the detailed description of the respective healthcare system context. The structure of the healthcare system and funding models were often not reported in the primary qualitative and mixed-methods studies. Moreover, the screening guidelines, and screening provision system might change overtime, which make a challenge to comprehensively include in this review article. We have included it as the limitation of the manuscript as follow;

"Moreover, the papers in the review lacked detailed description of contextual factors such as national or regional health system characteristics, funding models, and the presence of organized screening programs. Health system structures and screening recommendation guidelines may have changed over time since the publication of papers in the review. Therefore, the transferability of findings to different healthcare systems and settings should be interpreted with this potential limitation in mind." (Line: 457-463).

3. The barriers and enablers identified do not seem to be much different from those that have been described previously in the literature so I am unsure what these findings add as currently presented.

Response: Thank you for the thoughtful comment. While some barriers and facilitators have been reported in previous studies, this review contributes by synthesising all the available existing primary studies. The review provides a deeper understanding of PCDs' perceptions and experiences across the studies. Moreover, the evidence gap in low- and middle-income countries and focus on breast cancer screening was highlighted based on the findings of this review. These contributions have now been emphasised more clearly in the Discussion section. (Line: 440-444)

VERSION 2 - REVIEW

**Reviewer**	**1**
**Name**	**Cithambaram, Kumaresan**
**Affiliation**	**Technological University of the Shannon**

Date 20-Apr-2026

COI

The editor will contact you with a decision.

Reviewer 2

Name Sussman, Jonathan

Affiliation McMaster University, Division of Radiation Oncology

Date 14-Apr-2026

COI

---

authors have addressed comments
